# Serologic biomarker discovery for differentiating Lyme disease from diseases with similar clinical symptoms using broad profiling of antibody binding

**DOI:** 10.3389/fimmu.2025.1528524

**Published:** 2025-05-16

**Authors:** Tingting Zhang, Laurie Baert, Neal W. Woodbury, Laimonas Kelbauskas

**Affiliations:** ^1^ Biodesign Institute, Arizona State University, Tempe, AZ, United States; ^2^ Department of Immunology, Mayo Clinic, Scottsdale, AZ, United States; ^3^ Biomorph Technologies, Chandler, AZ, United States

**Keywords:** humoral immune response, Lyme disease, differentiating diagnosis, machine learning, peptide array analyses, seronegative Lyme disease

## Abstract

**Introduction:**

Lyme disease (LD) is a tick-borne disease that is a substantial public health burden with estimated about 0.5 million new cases per year in the US and increasing incidence. Differentiating Lyme disease, especially in its early stages, from other febrile illnesses with similar clinical symptoms (look-alike diseases) represents a significant challenge due to the lack of diagnostic tools. Current diagnostic tools based on serology were not specifically developed for differential diagnosis and show limited sensitivity in early LD resulting in high false negative rates.

**Methods:**

The work presented here focuses on a broad profiling of the humoral immune response in terms of circulating antibody repertoire in patients diagnosed with LD and a number of diseases with similar clinical symptoms. A combination of antibody binding to a library of linear, diverse peptides and machine learning methods revealed a panel of biomarker proteins from the proteome of the Borrelia burgdorferi bacterium (LD causing pathogen) that can be used to differentiate between LD and other diseases.

**Results:**

A subset of the biomarkers was independently validated and demonstrated to show robust differentiating power. Importantly, the discovered biomarkers distinguish between LD patients that previously tested negative with the current test standard (false negatives) and the look-alike diseases.

**Discussion:**

These findings are important in that the discovered biomarkers can be utilized for differential diagnosis of LD. Furthermore, because the discovery approach is agnostic, the results suggest that it can also be used for biomarker discovery of other diseases.

## Introduction

Tick-borne diseases (TBDs) have become a major public health challenge with a projected incidence rate of >35% of the global population by 2050 (1). Lyme disease (LD) is the most prevalent tick-borne zoonotic disease in the USA with an estimated 476,000 new cases each year and increasing incidence (2). Despite recent advances, the diagnosis of LD, especially in the early stages of the disease, remains challenging due to the limitations of the current testing methods. The current standard for LD diagnosis is based on the detection of antibodies raised by the humoral arm of the human immune system against specific antigens from the *Borrelia burgdorferi* proteome. These tests are only ~30% sensitive in the early stages of the disease at 96% specificity (3). Adding to the diagnostic challenge is the fact that LD typically presents with symptoms such as fever, muscle pain, and fatigue, which are shared with a number of other common diseases like the flu or seasonal cold. The “bullseye rash” [erythema migrans (EM)], a typical tell-tale sign of LD, does not appear in a substantial portion of patients or presents with differing morphology, further complicating diagnosis (4–6). Furthermore, there is increasing evidence that an EM-like rash can be present in patients after exposure to pathogens other than members of the *B. burgdorferi sensu lato* complex (7, 8). As a result, misdiagnosis with another disease can result in poor treatment outcomes.

A *B. burgdorferi* infection can involve a range of organs resulting in dermatological, cardiac, neurological, and musculoskeletal disorders. Successful differential diagnosis of Lyme disease against diseases with look-alike symptoms is required for timely treatment when antibiotics are most effective (9). Delays in diagnosis in approximately 40% of patients result from an absence of an EM rash, unnoticed tick bite, human factors, and confounding symptoms that indicate another disease (10). Other TBDs, such as *Babesia microti* and *Ehrlichia* spp., are spread by the same tick as *B. burgdorferi* and result in febrile illness with similar symptoms (11). One problem with the current diagnostic is that it was developed to differentiate LD from healthy controls. However, due to the significant symptom overlap with other, common diseases, the development of a diagnostic approach designed to distinguish between LD and a broad range of diseases would be very beneficial.

Lyme disease can be misdiagnosed as influenza, Epstein–Barr virus (EBV), and parvovirus B19, which cause similar fever, myalgias, and fatigue (12, 13). Cross-reactivity of antibodies raised during EBV and syphilis and against autoimmune markers on current serologic tests further confounds diagnostic tests (14–17).

*B. burgdorferi* presents unique challenges in even the acute disease presentation that are associated with its innate ability to modulate host immune system response (18). It is a highly antigenically heterogenic genospecies with 25 known serotypes of Outer Surface Protein C (OspC) alone (19). In addition, different strains carry different combinations of extra-genomic plasmids that encode immunogenic antigens (20, 21); recombinant antigenic variation alters the VlsE surface protein, which enhances immune evasion (22–24); there is geographic variation in antigens recognized by serum antibodies (25, 26); and co-infection can occur with multiple *B. burgdorferi* strains (27, 28) or with other tick-borne diseases such as *Babesia* (28, 29). Within the *B. burgdorferi sensu lato* complex, five genospecies including *B. burgdorferi*, *Borrelia afzelii*, and *Borrelia garinii* (with the latter two mainly found in Europe) are known to cause LD in humans (30), while others such as *Borrelia mayonii* cause an LD-like illness, and *Borrelia miyamotoi*, a relapsing fever spirochete, circulates via the same tick (31, 32). Direct detection methods targeting *B. burgdorferi* are limited due to the low bacterial load in the blood after the initial infection (33). Therefore, serology has been the method of choice for LD diagnosis based on the presence of antibodies specific to targets in the *B. burgdorferi* proteome.

This study was designed to address the question of whether a broad, agnostic profiling of the humoral immune response can identify a set of immunogenic proteins from the *B. burgdorferi* proteome that gives rise to antibodies with differential reactivity between LD and a number of other febrile diseases. Because LD is known to be highly heterogeneous in terms of the adaptive immune response of the host, as well as timing for disease progression and symptom severity, we utilized a method for broad and unbiased profiling of the circulating antibody binding repertoire in sera obtained from LD patients and from individuals diagnosed with other diseases that have similar clinical symptoms [look-alike diseases (LADs)].

To accomplish this, a random and sparse sampling of the entire combinatorial sequence space of short (6–13 amino acids long) linear peptides was represented on a peptide array consisting of 126,051 unique, randomly designed peptides that do not represent any specific antigen or pathogen. After exposing the peptides to antibodies contained in a serum sample, the binding profile of each patient’s antibodies to the peptide library is measured using a fluorescently labeled secondary polyclonal anti-IgG antibody, and this is read out as fluorescence intensity. A comprehensive sequence-binding relationship between the peptide array sequences and the measured IgG binding values is developed by training a machine learning (ML) algorithm. This model is then used to predict the total IgG binding in each serum sample to the proteins that make up the *B. burgdorferi* proteome or to the proteomes of other pathogens.

A number of candidate proteins from the *B. burgdorferi* proteome with predicted differential Ab reactivity are selected based on the predicted binding values. After selection, the candidate proteins were expressed in *Escherichia coli*, and their ability to differentiate between LD and look-alike diseases was evaluated on an orthogonal, bead-based assay.

Previous work from this lab (34–41) has demonstrated the utility of this approach to identify linear epitopes of a number of monoclonal antibodies (42), differentiate among different infectious diseases (43), and reveal substantial person-to-person variability in humoral immune response in LD patients (36). These studies have shown that despite the inherent limitation of the linear peptides in identifying conformational (discontinuous) epitopes, the method is capable of providing biologically relevant insight into humoral immune responses by broadly characterizing antibody binding profiles in a disease-agnostic manner. Importantly, the investigation of LD humoral immune response also revealed a differential humoral response between the seropositive and seronegative LD cohorts, suggesting that the two patient subgroups should be considered distinct. The same study also reported strong similarity in antibody binding profiles between LD and healthy controls from geographies endemic to LD, but not other locations, indicating potentially high seroprevalence in areas with high LD incidence.

In comparison with the previous work, the current study presented here is based on a substantially expanded LD+ cohort as well as expanded look-alike disease cohorts to increase the statistical power and generalizability of the approach in identifying immunogenic targets in LD. In principle, this type of approach opens the door to the discovery of novel, potentially more potent biomarkers for disease diagnosis and provides a disease-agnostic means for the identification of new therapeutic targets for a number of infectious and autoimmune diseases for which currently no cure exists.

Here, the approach described above was used to select candidate biomarker proteins from the *B. burgdorferi* proteome that show predicted differentiation between LD patients and patients with a range of other febrile diseases that have similar symptoms, resulting in substantial improvement in the sensitivity of the assay over the current serologic test standard. This involved antibody profiling on peptide arrays, predicted binding to the entire *B. burgdorferi* proteome, and a subsequent candidate biomarker selection process, followed by a validation of the selected targets.

## Results

### Antibody binding profiles measured on peptide arrays

Antibody binding of a total of 536 human serum samples representing seropositive (LD+) and seronegative (LD−) Lyme disease and 13 diseases with symptoms similar to LD (LADs; see **Table 1** for cohort breakdown) were profiled on the peptide arrays. The “seropositive” designation was assigned to the LD patients who presented with a rash of >5-cm diameter and tested positive with the Centers for Disease Control and Prevention (CDC)-recommended serologic test. Lyme disease patients who presented with a rash of >5-cm diameter but tested negative with standard two-tier test (STTT) were categorized as “seronegative” LD.

**Table 1:** Discovery cohort breakdown.

Disease	Number of samples
Seropositive LD (LD+)	186
Seronegative LD (LD−)	102
Alcoholic liver disease	9
Antinuclear antibodies	15
*Babesia*	23
Chlamydia	12
Dengue	3
Epstein–Barr virus	82
Fibromyalgia	1
Influenza	27
Mononucleosis	2
Parvovirus	9
Rheumatoid arthritis	14
Syphilis	19
West Nile virus	17
Total	521

Anti-SSA, anti-Sjogren’s syndrome-related antigen A autoantibodies; LD, Lyme disease.

The sequences of the peptides on the array were designed randomly with a goal of equally representing 19 (cysteine was excluded due to synthesis constraints) canonical amino acids on the array. As a result, the peptide arrays can be understood as an unbiased and agnostic way to evenly interrogate the binding of the patient’s antibody repertoire to the entire combinatorial space of 10-mer peptides. Due to the random and agnostic peptide array design, any differences observed in antibody binding between the study cohorts can be attributed to a specific humoral immune system response and should not be affected by the peptide array design per se.

**Figure 1:**
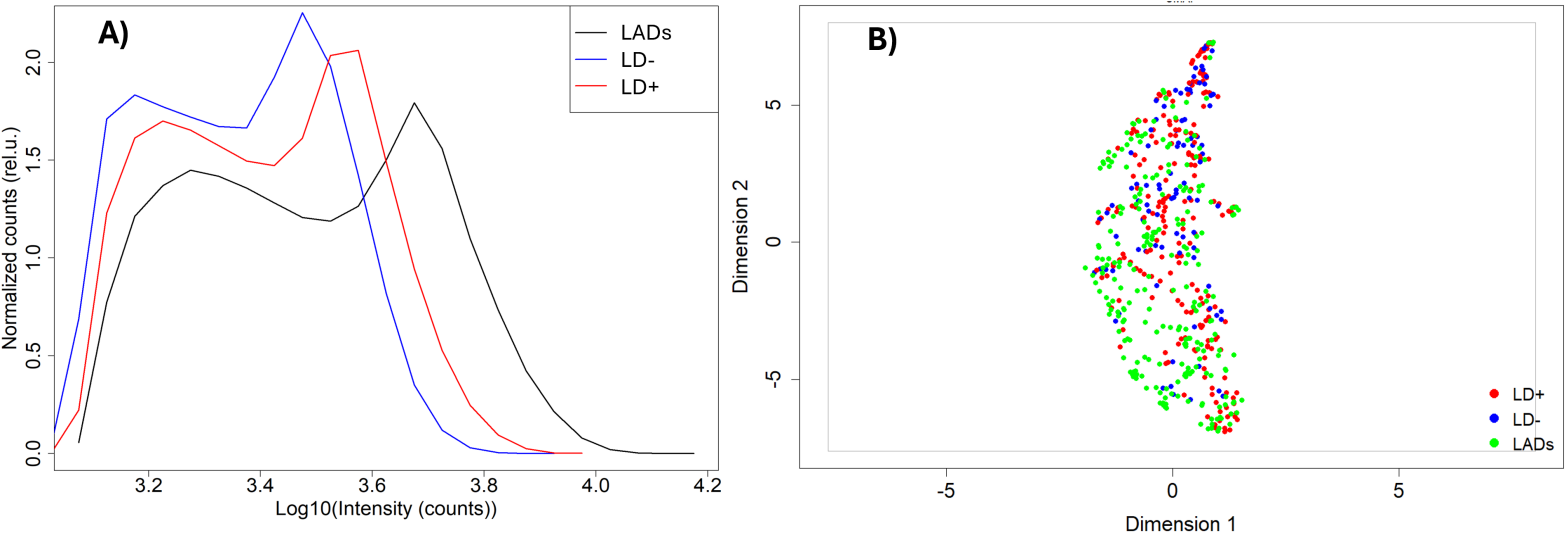
Comparison of antibody binding to library peptides between the three cohorts. **(A)** Intensity distributions of binding to the peptide library on the microarray. The intensity values have been log-transformed to make them more normal-like. The Y axis represents density of counts. **(B)** UMAP representation of the data shown in panel A with look-alike diseases, LD−, and LD+ represented as green, blue, and red circles, respectively. The dashed ovals represent three clusters within the distribution. UMAP, Uniform Manifold Approximation and Projection; LD, Lyme disease.

The distribution of binding intensities of total serum IgG to peptide array sequences shows an overall stronger binding for samples from the patient cohort with LADs compared to the LD+ and LD− disease cohorts (**Figure 1**). All three cohorts show bimodal distributions with two distinct peaks in their binding intensity. The first peak is centered at the low end of the binding intensity range close to the background binding signal on the array. Thus, the first peak likely represents the fraction of peptides that bind antibodies non-specifically and only weakly or not at all. The second peak in all three cohorts involves stronger binding peptide sequences. Despite the similar shape of the distributions between the three cohorts, the distributions show differences. The shift of the second peak with respect to the first varies markedly with cohort. The look-alike cohort shows the largest separation between the peaks with approximately 4× higher intensity of the second peak (0.6 on the log10 scale). The seronegative LD (LD−) group of patients exhibits the smallest ratio of ~1.6× in the shift between the two peaks, with the seropositive (LD+) cohort showing an intermediate shift of ~2×. The position of the second peak presumably captures the more specific binding and thus likely contains the disease-specific response. This suggests that antibodies in the look-alike disease group show an overall stronger immune response compared to both LD cohorts. Consistent with this, the LD+ cohort where clear antibody reactivity has been measured results in overall stronger binding than the LD− cohort, presumably reflecting the larger number of reactive antibodies present (**Figure 1A**).

The binding intensities between the three cohorts were qualitatively compared using the Uniform Manifold Approximation and Projection (UMAP) method for data dimensionality reduction and visualization (**Figure 1B**). The three disease cohorts show substantial overlap with one another in this representation (also see **Supplementary Figure 1** for pairwise comparison using UMAP; the look-alike and LD− cohorts show partial separation in a binary comparison; **Supplementary Figure 1B**). This suggests that despite the observed differences in the binding intensity distributions between the cohorts, differentiation based on the binding intensities of the individual peptide is not as clear. Interestingly, the UMAP representation suggests the presence of at least three subclusters in the data (**Figure 1B**), indicating underlying additional complexity with differences in fractional distributions among the cohorts. For example, cluster 3 contained only 27 out of 235 (11%) of the LAD patients, with the rest of the cohort distributed approximately evenly between clusters 1 and 2. In contrast, 39 out of 93 (42%) LD− individuals were included in cluster 3, while 17 (18%) and 37 (40%) were contained in clusters 1 and 2, respectively. The LD+ patients are distributed approximately equally among the three clusters.

**Figure 2:**
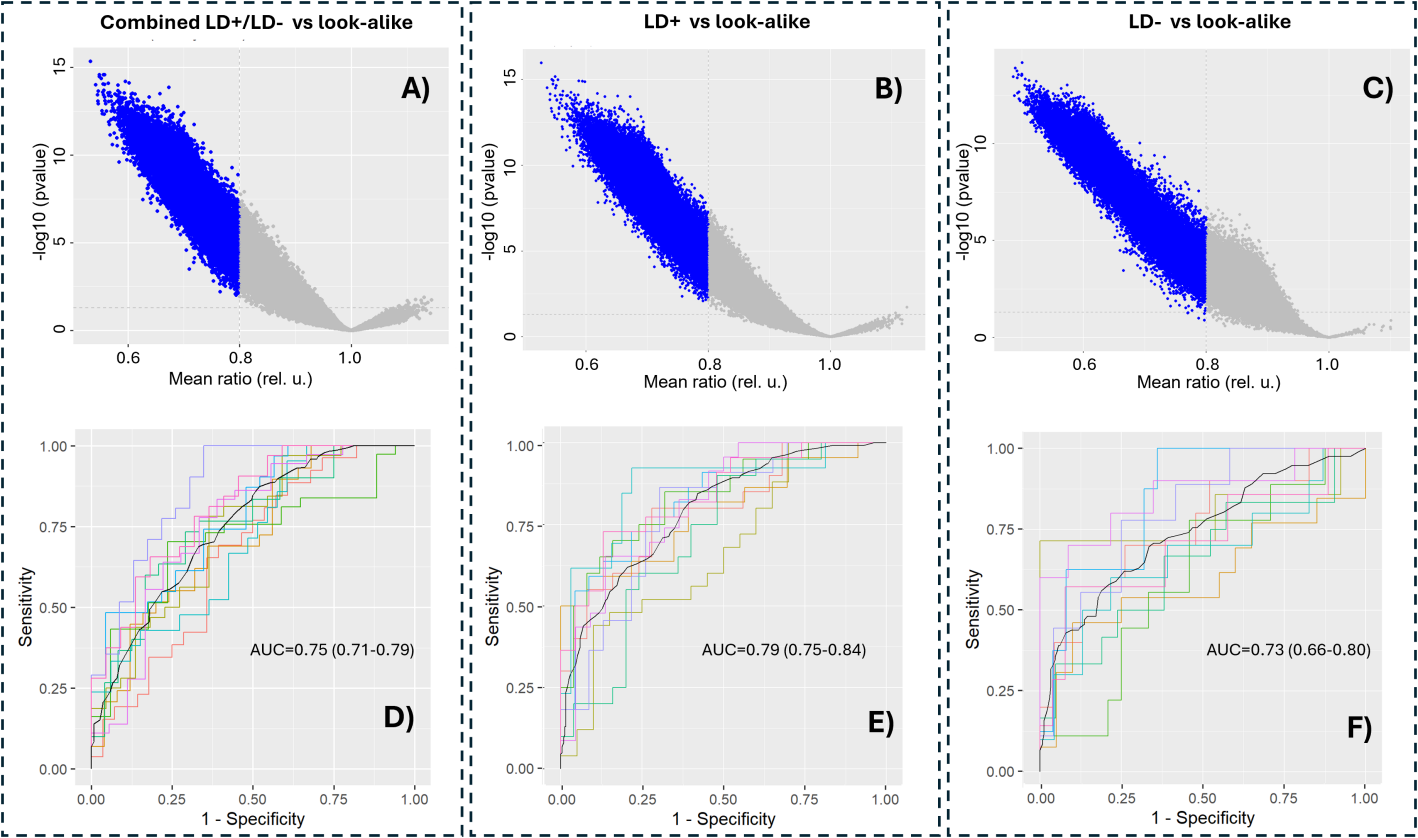
Separating LD+ and LD− cohorts provides better classification performance compared to the combined LD+/LD− cohort from the look-alike diseases. p-Value distributions (volcano plots; **(A–C)**) and classification performance in terms of receiver operating characteristic (ROC) curves **(D–F)** of the following contrasts between the cohorts: combined LD+/LD− vs. look-alike diseases **(A, D)**, LD+ vs. look-alike diseases **(B, E)**, and LD− vs. look-alike diseases **(C, F)**. In all three cases, an XGBoost classifier was trained on 90% randomly chosen peptides in the entire library (n = 126,051 peptides). The remaining 10% of the data were used for cross-validation, which was performed 10 times. The AUC values and their 95% confidence intervals are shown in the graphs. LD, Lyme disease; XGBoost, Extreme Gradient Boosting.

Because of the differences observed between the seropositive and seronegative LD groups compared with the look-alike diseases, the combined LD (LD+/LD−) cohorts were compared with the look-alike disease cohort in determining p-value distributions and building classifier models. As observed earlier when analyzing the binding intensity distributions (**Figure 1**), the majority of the peptides showed lower binding intensities in the LD cohorts compared with the look-alike diseases regardless of whether the LD cohorts were combined or not (**Figures 2A–C**). A comparison of classification performance using the Extreme Gradient Boosting (XGBoost) algorithm (**Figures 2D–F**) showed higher Area under the curve (AUC) values for both LD+ vs. LADs and LD− vs. LADs (AUC = 0.83, 95% CI: 0.77–0.98, and AUC = 0.85, 95% CI: 0.81–0.89, respectively) compared to the combined (LD+/LD−) vs. LADs [AUC = 0.77 (0.72–0.82)]. This result indicates a somewhat better classification in terms of AUC value when the two LD cohorts are considered separately. The classification results imply that the LD+ and LD− cohorts differ not only in terms of known biomarkers being positive in LD+ but also in terms of antibody reactivity that is unique to LD−.

### Predicted antibody binding to the *B. burgdorferi* proteome

**Figure 3:**
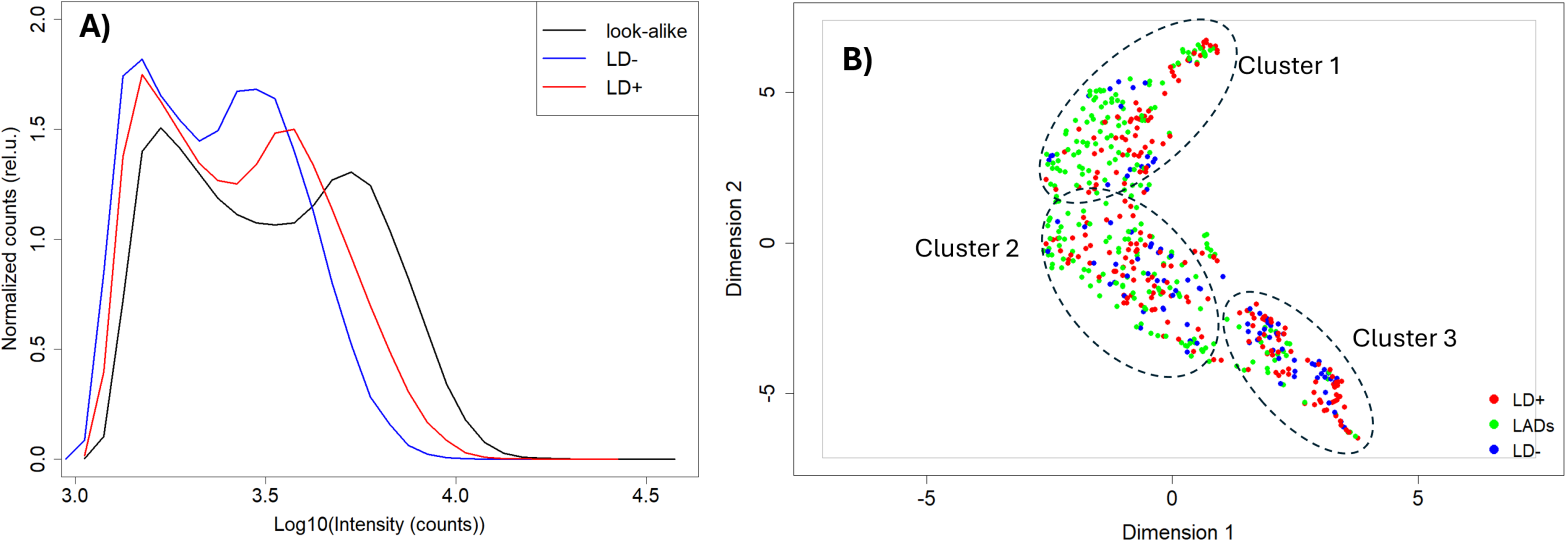
Mapping peptide array binding data onto the *Borrelia burgdorferi* proteome. **(A)** Predicted binding intensity distributions. **(B)** UMAP representation of the predicted Ab binding intensities to the tiled *B*. *burgdorferi* proteome with look-alike diseases, LD−, and LD+ represented as green, blue, and red circles, respectively. The distributions and the UMAP representation of the predictions show close similarity to the measured data. LD, Lyme disease; XGBoost, Extreme Gradient Boosting; UMAP, Uniform Manifold Approximation and Projection.

Due to the random nature of the peptide sequences in the array library, one cannot directly use the information about the sequence-binding relationship to determine what proteins from the *B. burgdorferi* proteome may be immunogenic and serve as potential candidate biomarkers. To select proteins from the *B. burgdorferi* proteome for further validation, ML approaches were used to model the underlying sequence-binding patterns and then project the patterns onto all *B. burgdorferi* proteins. The sequences of each protein from the *B. burgdorferi* B31 strain proteome (n = 1,219) were split into 10 AA-long tiles with nine amino acids (AA) overlapping between adjacent tiles. The proteome tiles were then one-hot encoded and used as input for the neural network (NN) models trained on the peptide array binding data of each sample, resulting in predicted binding intensities of every tile. These were assembled into a predicted binding map of each protein. Note that one separate model was trained on the binding data from each patient, resulting in *B. burgdorferi* binding predictions generated for each patient. A comparison of the predicted binding distributions (**Figure 3A**) shows similar characteristics to the measured binding on the peptide array. All three cohorts show distinct bimodal shapes with the first peak representing weak binders and the second peak capturing mainly the stronger interactions. With respect to the LD+ and LD− cohorts, the LAD group shows the largest separation between the two peaks. It is also shifted most toward the higher intensities (stronger interactions) compared with the other two cohorts, followed by the LD+ and LD− groups. However, there is one substantial difference between the measured and predicted distributions. The second peak (strong interactions) in the distribution of predicted values to the *B. burgdorferi* proteome is higher than the first peak (**Figure 3A**), in contrast to that observed in the measured array peptide binding values (**Figure 1A**). This suggests that the sequences from the *B. burgdorferi* proteome overall contain more peptides (tiles) that resemble antigenic targets of antibodies in each patient. Interestingly, a UMAP representation of the predictions (**Figure 3B**) revealed a distribution with fewer distinct subclusters as compared to the measured intensities (**Figure 1B**) even though the shape and overall overlap between the distributions of the three cohorts are similar. In addition, the LAD and LD− cohorts (green and blue dots in **Figure 1B**) appear to be separated more than observed in the measured data (**Figure 1B**), at least visually. Nevertheless, the overall similarity between the measured and predicted distributions indicates reliable model performance with regard to projecting patterns in the learned data onto a biologically relevant context.

### Statistical significance and classification performance using binding prediction to the *B. burgdorferi* proteome

**Figure 4:**
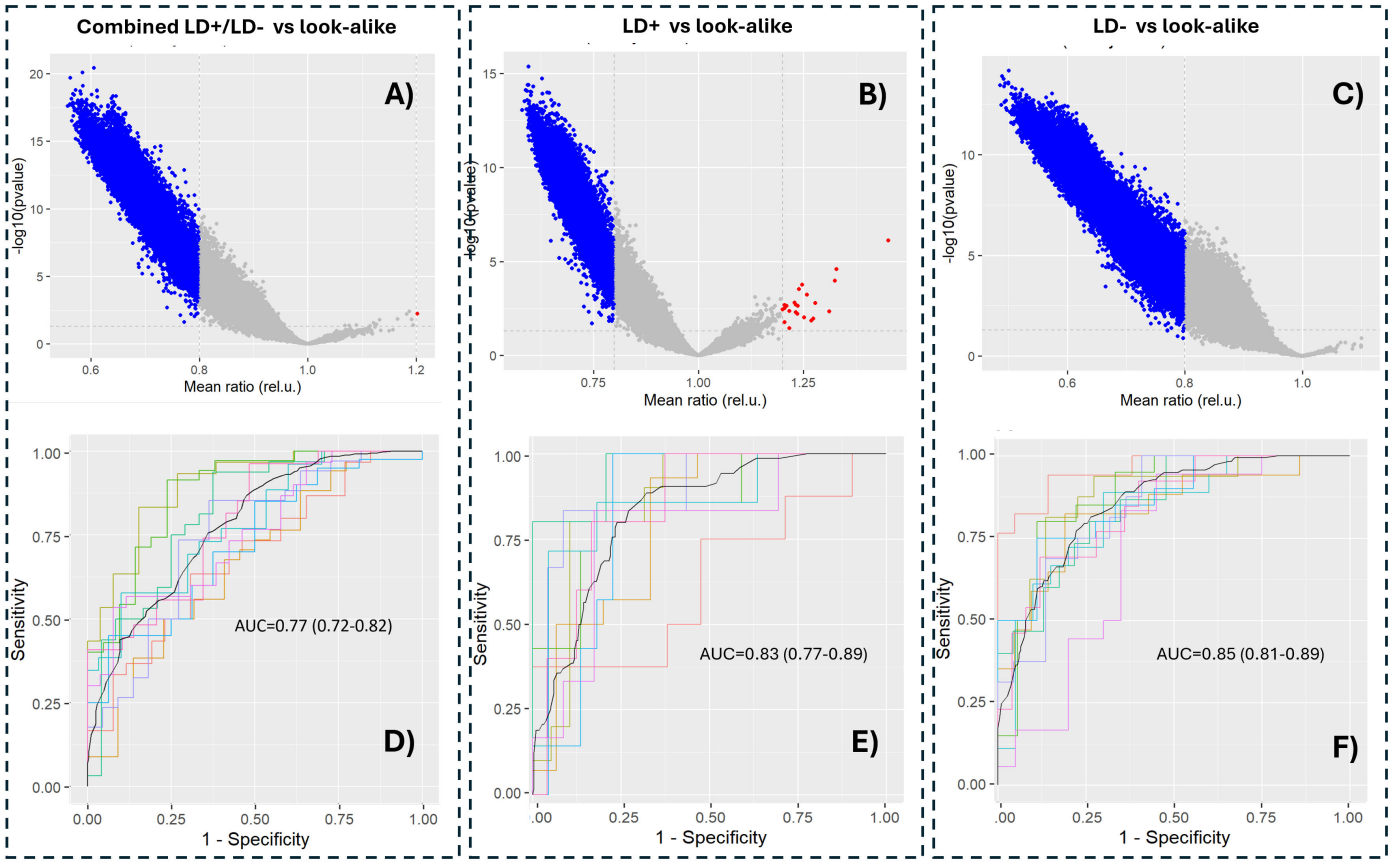
Statistical significance of predicted antibody binding intensities to tiles of the *Borrelia burgdorferi* proteome **(A–C)** and classification performance between the corresponding cohorts **(D–F)**. The horizontal axis in **(A–C)** represents the ratio of intensity means between the corresponding LD cohort and the LAD. The p-values were calculated using a t-test and are not adjusted for multiple hypothesis comparison to better highlight differences among the comparisons. The blue dots in **(A–C)** depict tiles with predicted binding intensities below a threshold intensity ratio of 0.8 between the LD and LAD cohorts. LD, Lyme disease; LAD, look-alike disease.

The model predictions of antibody binding to the *B. burgdorferi* proteome were analyzed in terms of p-value distributions (**Figures 4A–C**). Similar distribution characteristics were observed to those of the data measured on the peptide arrays (**Figure 2**), with the majority of the proteome tiles showing lower predicted binding intensities to antibodies in sera of the LD+ and LD− cohorts compared to the LAD group of patients. Two arbitrary threshold ratio values of 0.8 and 1.2 were used to better highlight trends in the differential binding profiles. There are relatively few tiles that show stronger binding in the LD cohort than in the LAD cohort, consistent with the binding distributions for the cohorts (**Figure 3A**). Classifier models were trained on the predicted *B. burgdorferi* binding values (**Figures 4D–F**). Lower classification performance was observed compared to models trained on the measured peptide array data (**Figures 2D–F**). All three classifiers showed similar AUC values, suggesting, in contrast to the results obtained with the peptide array data, comparable differentiation between the two LD and the LAD cohorts. Unlike the models trained on the measured peptide array data, the classification of the combined LD+/LD− vs. LAD cohorts was similar to the classification using the separate cohorts.

### Candidate protein biomarker selection from the *B. burgdorferi* proteome

Note that the analysis of predicted total IgG binding to the *B. burgdorferi* proteome, as described above, was performed at the level of separate sequence tiles and not complete proteins. This resulted in generally low effect sizes and mediocre classification performance, suggesting that there are no strong candidate biomarkers at the individual sequence tile level.

It is not very surprising that short sequence pieces of potential antigens do not provide strong differentiation. Past work has shown that the immune response to Lyme disease is very heterogeneous (36, 44), and thus, even if patients have antibodies against common proteins, they may well not bind to the same epitopes in those proteins. An alternative is to consider the aggregate predicted binding to each protein in the proteome and in this way select candidate protein biomarkers from the *B. burgdorferi* proteome with high predicted differentiating power between the combined LD+ and LD− cohorts and the LAD group of patients. Two different complementary methods were used for candidate protein selection. The first method was based on ranking the proteins by the lowest p-value determined from the predicted binding values of the tiles that make up its sequence. The p-values were calculated using the outlier sum statistics (45) (see Materials and Methods) selecting the proteins with a false discovery rate (FDR) of <0.05. The outlier sum statistics method was chosen to account for the long tails in the binding intensity distributions observed on the peptide array assays. It has been demonstrated that outlier sum statistics outperforms the t-test method for calculating the statistical significance of distributions with outliers (45). This method of selection resulted in a total of 19 protein candidates being selected from a total of 1,281 proteins (**Supplementary Data Sheet 1**) contained in the *B. burgdorferi* proteome (**Table 2**) (Equations 1–4). The last two proteins in **Table 2** were included because the FDR values were above the 0.05 cutoff by only a small margin.

**Table 2:** Selected candidate biomarker proteins from the *Borrelia burgdorferi* proteome using the outlier sum statistics method.

UniProt ID	p-Value	FDR	Protein
O51353	2.92E−06	3.69E−03	50S ribosomal protein L1
O51555	9.42E−06	5.60E−03	Trigger factor
O51247	1.33E−05	5.60E−03	50S ribosomal protein L31 type B
P52323	2.13E−05	6.34E−03	RNA polymerase sigma factor RpoD
O51757	2.51E−05	6.34E−03	UDP-*N*-acetylmuramate–l-alanine ligase
O51112	3.92E−05	8.27E−03	Uncharacterized protein BB_0085
O50893	1.45E−04	2.42E−02	HTH_OrfB_IS605 domain-containing protein
O51178	1.68E−04	2.42E−02	Uncharacterized protein
O51143	1.94E−04	2.42E−02	Pts system, maltose and glucose-specific IIABC component
O51632	1.77E−04	2.42E−02	Uncharacterized protein BB_0689
O51604	2.10E−04	2.42E−02	GTPase Era
O51141	2.61E−04	2.53E−02	Single-stranded DNA-binding protein
O51560	2.42E−04	2.53E−02	30S ribosomal protein S4
O51401	2.80E−04	2.53E−02	Fructose-bisphosphate aldolase
O51286	3.10E−04	2.55E−02	Ribosomal RNA small subunit methyltransferase H
O50821	3.23E−04	2.55E−02	Adenine deaminase
O51324	5.45E−04	4.05E−02	Uncharacterized protein
O50667	7.31E−04	5.14E−02	Type I restriction enzyme r protein n terminus (Hsdr_n)
P53362	8.00E−04	5.32E−02	tRNA uridine 5-carboxymethylaminomethyl modification enzyme MnmG

p-Values are not adjusted for multiple comparisons. FDR is p-value corrected for multiple comparisons using the Benjamini–Hochberg method.

FDR, false discovery rate.

The second method for candidate protein selection utilizes the XGBoost classifier trained on the predicted binding intensities comparing the combined LD cohorts vs. the LAD cohort (**Figure 4A**). The XGBoost algorithm is based on a decision tree structure and intrinsically performs feature selection during training. As a result, a trained classifier is based on a subset of the features (protein tiles, in this case) that contribute to the classification of the two cohorts. To identify protein candidates, first, only the tiles that were selected by the algorithm in all the cross-validation rounds (n = 10) were kept. Second, the *B. burgdorferi* proteins were then ranked by the number of different tiles they contained that met this criterion (**Supplementary Data Sheet 2**). Proteins that contained at least three tiles from the list above were selected as candidate biomarkers, resulting in a total of 34 proteins (**Table 3**). The arbitrary threshold for the number of tiles per protein was set to 3 to both limit the number of candidate proteins and increase the likelihood of a protein showing true differentiating power.

**Table 3:** Candidate biomarker proteins selected utilizing the classifier-based method.

UniProt ID	Tile count	Protein
O51465	5	Uncharacterized protein
O51735	5	Outer membrane protein
O51067	4	Uncharacterized protein BB_0038
O51157	4	Transcription elongation factor GreA
O51291	4	NAD kinase
O51578	4	RecBCD enzyme subunit RecB
P42555	4	Chaperone protein HtpG
O51319	4	DNA helicase
O51409	4	Transporter, small conductance mechanosensitive ion channel (MscS) family
O51504	4	Uncharacterized protein
O51195	3	Uncharacterized protein BB_0173
O51229	3	DNA mismatch repair protein MutL
O51316	3	Aspartyl/glutamyl-tRNA(Asn/Gln) amidotransferase subunit B
O51349	3	DNA-directed RNA polymerase subunit beta
O51540	3	Arginine–tRNA ligase
O51560	3	30S ribosomal protein S4
O51680	3	Valine–tRNA ligase
P50062	3	Elongation factor Tu
P70838	3	Uncharacterized protein BBD11
Q44737	3	Chemotaxis protein CheA
G5IXI8	3	Uncharacterized protein
H7C7M1	3	ErpM protein
O51310	3	Oligopeptide transport system permease protein OppC
O51326	3	Uncharacterized protein
O51381	3	Sensory transduction histidine kinase, putative
O51485	3	Uncharacterized protein
O51570	3	*N*-acetylmuramoyl-L-alanine amidase, putative
O51574	3	Pts system, fructose-specific IIABC component
O51655	3	zf-RING_7 domain-containing protein
O51687	3	Oligopeptide ABC transporter, permease protein
O51770	3	Exonuclease SbcC
O51774	3	ATP-dependent Clp protease, subunit C
O51784	3	Lipoprotein, putative
Q9RZW8	3	Adenine-specific DNA methyltransferase

Tile count indicates the number of tiles from the corresponding protein that were selected by the classifier in each cross-validation round (n = 10).

Interestingly, neither of the lists contains any of the serologic biomarkers used in the current LD testing standard. This indicates that the differential humoral immune response between the LD and LAD cohorts may have a different set of target antigens than when comparing LD with healthy controls (36). The two lists show no overlap and represent two unique sets of proteins. The list produced by method 2 does contain several proteins [including the two top-ranked proteins in **Table 3**: the outer membrane protein (O51735) and an uncharacterized protein (O51465)] that are either known to be located on the membrane of the bacterium or are predicted extracellular (secreted) proteins. The cellular location makes these proteins accessible to antibody binding and provides further support for biological inference of the protein selection method. The finding that neither of the two lists contains any of the known LD biomarkers, e.g., the VlsE protein, is likely due to the difference in cohorts being compared. The standard serologic biomarker panel was developed to differentiate between LD and healthy controls, whereas here, LD was compared with LADs. Furthermore, the finding that the LD+ and LD− groups of patients were characterized by distinct antibody reactivity profiles, as demonstrated previously (36) and in this study (**Figure 2**), means that combining the two cohorts as conducted has the potential to result in a set of biomarkers that better differentiates the combined LD cohort from LADs.

### Candidate biomarker verification using bead-based multiplex binding assays

**Figure 5:**
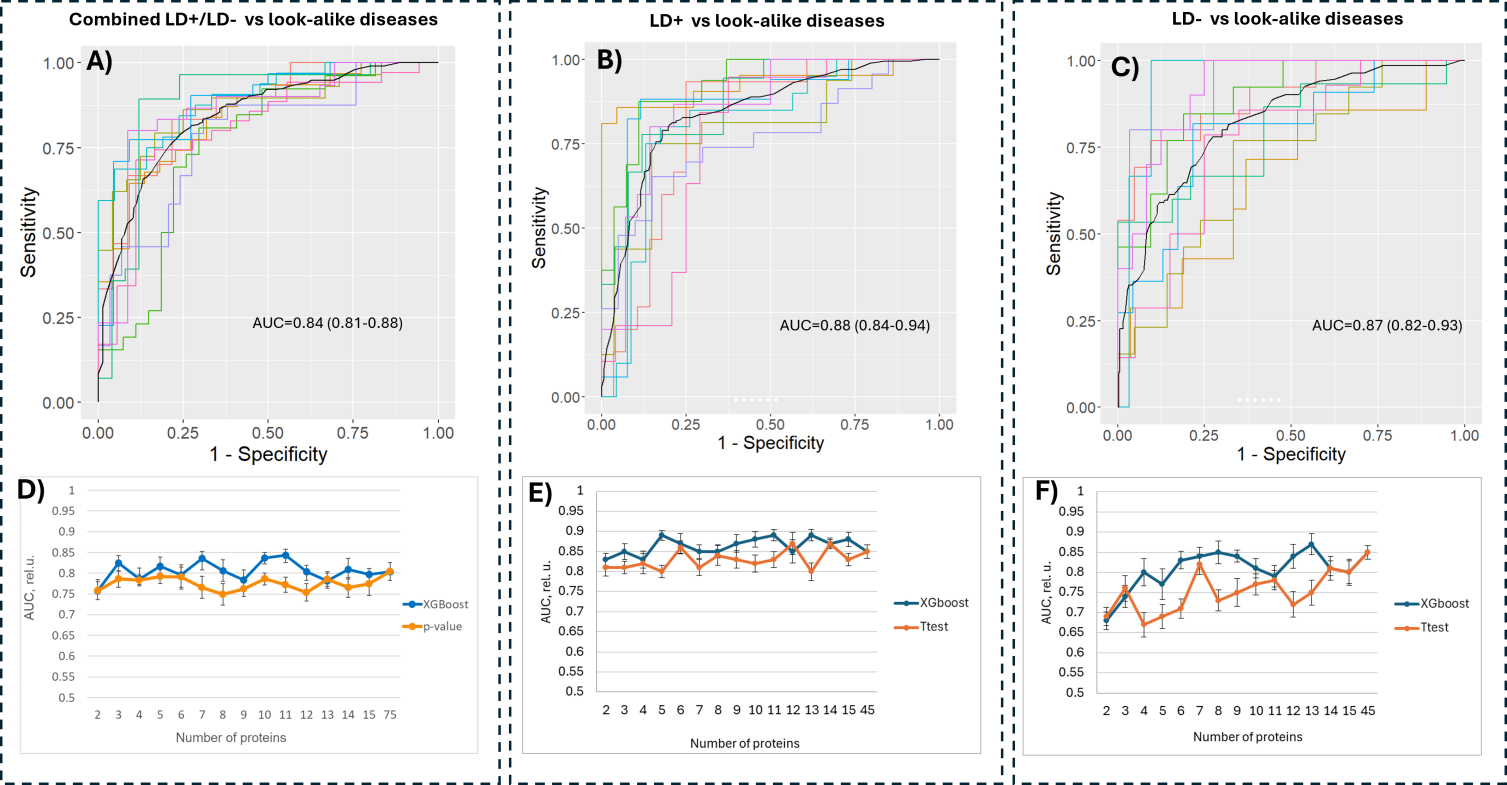
Classification performance of models trained on the bead-based assay data. Combined LD+/LD− **(A)**, LD+ **(B)** and LD− **(C)** cohorts were each compared against the sera from the patients in the LAD cohort using subsets of proteins that resulted in highest accuracy. The number of proteins used for training was varied for all three contrasts **(D–F)** by selecting a subset of biomarker candidates either based on predictive power calculated by a classifier trained on a full set of proteins (blue curve) or by ranking the proteins based on the p-value (orange curve). LD, Lyme disease.

A total of 53 candidate proteins from the *B. burgdorferi* proteome were selected for further consideration. However, only 44 of these were successfully synthesized; the nine remaining proteins were excluded from synthesis due to substantial transmembrane regions. In addition, the VlsE protein, a biomarker that is currently being used for standard serology testing for LD, was included as a positive control for the LD+ samples. For validation assays, the proteins were attached to carboxylated paramagnetic beads using *N*-hydroxysulfosuccinimide sodium salt (NHS) chemistry (Materials and Methods). The beads were incubated with serum samples, and the antibody binding was measured as fluorescence intensity using a secondary polyclonal anti-IgG antibody labeled with phycoerythrin. A total of 185 LD+, 102 LD−, and 236 LAD samples were used for validation with the bead-based assays (**Supplementary Table 1**). All samples were assayed in duplicate, and the mean values of the duplicates were used for further analysis. For data analysis, the binding values were converted to a log10 scale (**Supplementary Figure 3A**). The background binding signal was determined as the fluorescence intensity of the protein with the lowest coefficient of variation (CV) across all three cohorts. Low variation in the binding intensity of such a protein indicates that its binding is not disease-specific and can be used as a reference. While “blank” beads, i.e., beads not conjugated to any of the proteins, were also included in the assay as negative controls, it was observed that they showed some differential binding between the cohorts. It is possible that some of the antibodies in the three groups of patients exhibit preferential binding to the carboxyl moiety on the negative control beads. Further analyses, including classifier training, were performed with log-transformed intensity values that were used directly without further normalization. For classifier training, the LD+ and LD− cohorts were separated and contrasted against the LAD cohort individually. This was conducted based on the earlier findings in this study that suggested a different humoral immune response profile in the two groups of patients (**Figures 1**-**4**). One of the goals of the biomarker validation study was to determine if it would be possible to reduce the number of candidate proteins to a smaller subset to reduce the complexity of a potential diagnostic assay. To this end, classifier training was performed using all biomarkers and selected subsets. Feature selection was performed using two different methods: a) first, an XGBoost classifier was trained on data from all proteins. Because the algorithm performs feature selection during training, the proteins with the highest differentiation power (see **Supplementary Figure 3B** for a cohort-level comparison of two highest ranking by importance proteins) were used for training another XGBoost classifier on the reduced set of features. b) Proteins were selected based on the p-values of a t-test whereby a number of proteins ranked by increasing p-value (decreasing statistical significance) were chosen for classifier training. **Figures 5A–C** show the receiver operating characteristic (ROC) curves of classifiers trained using the XGBoost algorithm and a subset of biomarkers that resulted in the best classification accuracy (highest AUC value) in the entire range of the number of proteins selected for training. The classifiers were trained to distinguish between three different contrasts: a combined LD+/LD− cohort and LAD, LD+ vs. LAD, and LD− vs. LAD patients. Classification accuracy in terms of AUC value was determined as a function of the number of proteins included in the training dataset. A comparison of the best classification performance achieved for each contrast when varying number of proteins in the panel was slightly lower for the combined LD vs. LADs with AUC = 0.84 (95 CI: 0.81–0.88) (**Figure 5A**) than the two other contrasts with AUC values of 0.88 (95 CI: 0.84–0.94) (**Figure 5B**) and 0.87 (95 CI: 0.82–0.93) (**Figure 5C**) for LD+ vs. LAD and LD− vs. LAD contrast, respectively. Classification performance as a function of the number of features (proteins) used in training (**Figures 5C–E**) varies only slightly for the combined LD vs. LADs and LD+ vs. LADs. In comparison, the AUC values for the LD− vs. LAD differentiation showed a slight but notable upward trend with the increasing number of features (**Figure 5D**). This suggests that the differentiating information is distributed more broadly in the LD−/LAD than in the LD+/LAD contrast. A comparison of the AUC values obtained with the full set of proteins (n = 45) indicates that all three contrasts show a reduction in classification accuracy when the full set of proteins is used. Interestingly, the LD+ vs. LAD contrast shows improved performance with AUC = 0.88 (95 CI: 0.84–0.94) when using 11 proteins that rank highest by importance/differentiating power as determined by the classifier trained on the full set of proteins (**Figure 5A**). While the LD− vs. LAD classification gave a comparable performance to the LD+ vs. LAD classification when all features were used, there is one substantial difference between them. Interestingly, when comparing the biomarkers ranked by predictive power to distinguish between LD+/LADs and LD−/LADs, it is clear that panel composition differs substantially between the two comparisons (**Table 4**). Here, the gain parameter represents the fraction of overall classification performance that a particular protein biomarker contributes to the total classifier models shown in **Figures 5B, C**. The higher values represent stronger differentiating power with the gain values of all biomarkers used adding up to 1. As expected, VlsE (UniProt ID G5IXI6) exhibits by far the most differentiating power (0.37) in separating LD+ from LAD patients. Consequently, the removal of VlsE from the training dataset resulted in a marked drop in the AUC value to 0.71 (data not shown). However, it is also clear that VlsE alone was not sufficient to achieve the demonstrated classification performance (**Figure 5B**) and that additional biomarkers made smaller but substantial contributions to the classification performance. In contrast, VlsE had a distinguishing power of only 0.05 in the LD−/LAD contrast with three other proteins scoring higher. This is consistent with the LD− cohort being negative on standard panels containing VlsE. Overall, the data in **Table 4** suggest differences in biomarker panels between the two contrasts, with VlsE being the only protein shared among the 10 highest-ranking biomarkers.

**Table 4:** Comparison of first 10 biomarkers ranked by predictive power (gain) between LD+/LAD and LD−/LAD contrasts.

Protein number	LD+ vs. LADs		LD− vs. LADs	
	UniProt ID	Gain	UniProt ID	Gain
1	***G5IXI6(VIsE)***	***0.370383***	O51324	0.081201
2	O51784	0.045986	P50062	0.066731
3	H7C7M1	0.029262	O51286	0.057566
4	P53362	0.029069	***G5IXI6(VIsE)***	***0.05673***
5	O51291	0.026474	O51555	0.054708
6	O51353	0.026081	H7C7M1	0.04691
7	P52323	0.024759	O50667	0.045187
8	O51632	0.021474	O51229	0.0423
9	O51326	0.020214	P53362	0.041274
10	O51655	0.018787	O51570	0.031742

LD, Lyme disease; LAD, look-alike disease. The values in bold represent the VlsE protein, the main biomarker currently used in the standard LD serology.

Both feature selection methods (t-test and XGBoost) resulted in comparable classification outcomes, with the t-test-based selection method resulting in lower performance compared to the classifier-based method (**Figures 5C–E**). The classification performance as a function of the number of selected proteins demonstrates that one can substantially reduce the number of antigens in the panel without markedly affecting classification performance. For example, the data in **Figure 5D** show that one can achieve comparable performance for differentiating between the combined LD and LAD patients with as few as five proteins, whereas only slightly reduced performance can be reached with five and six proteins for LD+ vs. LADs (**Figure 5E**) and LD− vs. LADs (**Figure 5F**), respectively.

In summary, the validation assay data analysis suggests that one can accurately differentiate between the two LD cohorts and the look-alike diseases using a subset of protein biomarkers predicted to have differentiating power using the peptide array data. The most important finding is the fact that the LD− patients who tested previously negative in the standard serology test can be reliably differentiated from the LAD patients.

## Discussion

This study was designed to address the two following questions. First, in a general sense, can a broad, agnostic profiling of the humoral immune response using short, linear peptide libraries with randomly generated sequences that equally but sparsely sample an entire combinatorial space of peptides with the same length be utilized to extract biologically relevant information concerning immunogenic targets that the humoral immune system is responding to? The second, more specific question is focused on whether LD can be reliably differentiated from other diseases with similar clinical manifestations. Given the agnostic nature of the method and its unbiased approach to profiling circulating antibody binding, answering these questions would enable the evaluation of the method’s ability to identify novel diagnostic biomarkers. Such a method has the potential to be used for answering similar questions for a broad range of diseases.

The humoral immune response profiling in the three cohorts reveals a heterogeneous picture in terms of antibody binding patterns. A comparison of the binding intensity distributions shows that, overall, the lowest antibody reactivity is in the LD− group of patients as judged by the location of the second peak and the width of the distributions (**Figure 1A**). The substantially stronger response observed in the LD+ cohort may be due to a later timepoint in the acute LD stage when the samples were collected from these patients. The longer duration of immune system exposure to the pathogen in these patients may allow for a more robust adaptive immune response to be mounted against the bacterium, resulting in a stronger and more focused antibody response. It is also possible that the antibody response in LD− patients is a result of the immunosuppressive mechanisms intrinsic to *B. burgdorferi* that subdue or abrogate an early response and result in more time for the bacterium to establish an infection. In comparison, the LAD binding distribution characteristics suggest a substantially stronger response, despite the fact that this cohort encompasses a number of different diseases. This difference is consistent with the notion of the immunosuppressive role of the bacterium when interacting with the host immune system. Despite the observed differences in the binding intensity distributions, the array peptide binding patterns of the three cohorts are similar, as evidenced by the UMAP representation of the binding data (**Figure 1B**). This finding underscores the difficulty in distinguishing LD from other diseases due to large patient-to-patient variability in humoral immune response and possibly partial cross-reactivity between antibodies raised against the different pathogens. Cross-reactive antibodies have been identified to overlap with LD-specific responses in Epstein–Barr virus, *Treponema pallidum* infections (14–16), and rheumatoid arthritis (46–48). Previous publications from this lab and others have reported high levels of patient-to-patient variability in LD in terms of antibody reactivities (36, 44). The results presented above are based on serum samples collected from several biobanks potentially increasing the patient-to-patient variation further due to differences in sample collection and storage protocols, testing, and different geographic locations. The use of ML models trained on the peptide array binding data enabled the learned sequence-binding relationship to be “transferred” onto a biologically relevant level by predicting antibody binding to a tiled *B. burgdorferi* proteome. The predicted binding intensities exhibited similar distribution characteristics as the peptide array intensities (**Figure 3**), suggesting that binding pattern information as measured on the peptide array is properly captured on the proteome. Despite the somewhat reduced distinction among the hypothetical clusters of individuals observed in the binding data measured on the peptide arrays, the overall intensity distribution characteristics in terms of shape (**Figures 1A**, **3A**) and UMAP representation (**Figures 1B**, **3B**) between the measured and predicted data show close similarity. The binding predictions generated by NN models trained on each individual’s data showed high accuracy (**Supplementary Figure 2**), supporting the validity of the chosen predictive ML models. These data further suggest that compared to the LD+ cohort, the LD− cohort shows a distinct, although weaker, humoral response with possibly more pronounced person-to-person variability to the pathogen. The decreased overall binding is consistent with either being in the early stages of the infection or the inability to mount a response because of the immunosuppressive mechanisms engaged by the bacterium.

The observed similar classification performance between the measured and predicted binding values of models combining the LD+ and LD− cohorts (**Figures 2D**, **4D**) as opposed to the classifiers contrasting the two cohorts against the LAD patients separately (**Figures 2E, F**, **4E, F**, respectively) indicates that the differentiating power of the two classifiers is comparable. This result can likely be explained by the increased sample size in the combined LD cohort that leads to better classification model generalization and consistency that are less dependent on the source of the training data (measured vs. predicted). The finding further suggests the importance of having adequately sized patient cohorts in LD studies where patient-to-patient variability is notable and needs to be taken into account.

Importantly, the difference in the biomarker panels in terms of contributions to classification performance (**Table 4**) implies that the pathogen-specific antibody reactivity profile in LD− patients is different from that in the LD+ group. The only overlap between the two panels is the VlsE protein, yet its relative contributions to the overall differentiating power is approximately an order of magnitude less in the LD− cohort analysis. There is no other overlap among the nine remaining biomarkers. Also, the predictive power in the LD−/LAD contrast appears distributed more evenly among the panel biomarkers, suggesting a more dispersed humoral response in the LD− cohort compared to the LD+ cohort. The difference in the antibody reactivity profiles is also consistent with the negative standard serologic testing outcome for the LD− patients and may explain why no antibody reactivity to the biomarkers used in the standard serologic LD test is present. Nevertheless, despite the lack of antibody response to the current standard of testing, the findings of this study demonstrate that in these patients, there is an ongoing, *B. burgdorferi*-specific humoral immune response toward a set of immunogenic targets that are different from those typically found in seropositive LD patients using the current testing standard.

Interestingly, it was found that the classifiers trained on the predicted binding values of *B. burgdorferi* protein tiled sequences (linear 10 AA long peptides with 9 AA overlap) to distinguish between LD+ or LD− and LADs (**Figures 4E, D**) showed substantially lower differentiating accuracy than the classifiers trained on binding data obtained with full recombinant proteins in the bead-based assays (**Figures 5B, C**). Note that the proteins for the assays were selected based on the binding to the tiled proteins from the *B. burgdorferi* proteome. This suggests that the “hit” tiles identified in the analysis truly belong to proteins that are targeted by the humoral immune response. These tiles serve as linear proxies of binding to fully assembled proteins that, once expressed, show substantially stronger differential binding than the linear tiles due to the presence of full structural epitopes. One possible explanation is that a fraction of the linear protein tiles “hits” may contain short (3–6 amino acid long) motifs or their mimotopes (sequences that differ in amino acid arrangement but show similar physicochemical properties to the actual motif) that represent different linear portions of the same structural epitope(s) of a protein. If true, antibody binding to the fully assembled structural epitope on the protein would be substantially stronger and show a larger differential signal than the separate linear motifs of the peptides. The substantial increase in classification accuracy with full proteins therefore implies that at least some of the protein structure-specific binding is captured with the linear peptide arrays.

The fact that the peptide libraries are based on short peptides without any particular structural information is a major limitation given that the majority of peptide epitopes are structural and discontinuous. Nevertheless, earlier work from this group has demonstrated the utility of the approach to distinguish with high accuracy between a number of different diseases based simply on the binding patterns of antibodies contained in the blood (43). This study provides further support for the notion that some of the structural epitope information may be contained in the linear array binding data through the representation of the linear fragments that make up some of the structural epitopes. As a result, one may be able to utilize the binding to linear peptide data to identify immunogens containing either linear or a combination of linear and structural epitopes targeted by the immune system in response to pathogen infection.

In conclusion, the findings of this study highlight several challenges one is faced with when distinguishing LD from other diseases with similar clinical manifestations, especially in the early stages of LD. Strong patient-to-patient variability in the humoral immune response to *B. burgdorferi* combined with previously demonstrated cross-reactivity of antibodies raised in response to other pathogens both act as confounding factors in distinguishing LD from other LADs. Nevertheless, it was possible to identify and validate a panel of biomarkers that robustly differentiates between seropositive or seronegative LD and LADs. Furthermore, the results suggest a different humoral immune response profile in the LD+ and LD− patients through the finding of separate panels of biomarkers that are specific to each condition. Perhaps most importantly, the ability to distinguish between seronegative LD patients and LADs is especially valuable, as these patients would have been deemed non-LD and either misdiagnosed with another disease or subjected to further unnecessary testing. The study findings also corroborate the notion that combining data of polyclonal antibody binding to a library of linear peptides with machine learning models provides biologically relevant information about the humoral immune response underlying an acute infection with the *B. burgdorferi* bacterium. Due to the agnostic nature of the approach, it is also likely that the method can be utilized for profiling the humoral response and biomarker discovery for a number of other diseases with a strong humoral immune system involvement.

## Materials and methods

### Samples

Seropositive LD (LD+) patient serum samples were obtained from the Lyme Disease Biobank Foundation, Portland, OR (3), CDC, and several commercial biobanks (Boca Biolistics, Pompano Beach, FL; Discovery Life Sciences, Huntsville, AL; and SeraCare, Milford, MA). Samples were collected from patients with signs and symptoms of LD. Samples were tested using the STTT and categorized as seropositive Lyme disease having either an EM rash greater than 5 cm in diameter or PCR confirmation combined with positive STTT serology. The seronegative Lyme disease samples were obtained from patients having an EM rash greater than 5 cm in diameter, but without positive STTT serology, and were obtained from the Lyme Disease Biobank Foundation. These patients were diagnosed with Lyme disease by a physician based on the patients’ clinical symptoms. Participants were enrolled in East Hampton, NY, Central Wisconsin, and Martha’s Vineyard, MA. Each of the three cohorts contained equivalent numbers of patients from each collection site. Cohorts and collection sites were balanced across each assay batch of microarrays. The patient samples for the diseases with similar etiology to LD (look-alike diseases) were obtained from several commercial sources (Boca Biolistics, Pompano Beach, FL; Discovery Life Sciences, Huntsville, AL; Creative Testing Solutions, Tempe, AZ; and SeraCare, Milford, MA). Note that these samples were obtained from commercial biobanks, with no data about their previous exposure to LD provided. Given that the samples were collected outside of the LD endemic areas, it is unlikely that these patients have been exposed to *B. burgdorferi* infection.

### Peptide microarray assays

Peptide microarrays containing diverse peptides were synthesized in a commercial production facility (Cowper Sciences, Chandler, AZ), following a previously described library design and photo-lithography-based manufacturing process (34, 36, 37). Briefly, the microarrays used contained 126,051 diverse peptides plus a set of 6,203 control peptides of varying lengths ranging from 6 to 13– amino acids. The standard serum Ab profiling assay protocol described by Arvey et al. (34) was used and modified for a modular research use assay system as described by Kelbauskas et al. (36). Samples were thawed from single-use aliquots and diluted to 1:625 in assay buffer (Phosphate-buffered saline/Tween (PBST) with 0.05% Tween 20, 0.1% ProClin 950, and 1% mannitol). Diluted samples (90 μL) were applied to arrays and incubated for 1 h at 37°C with mixing (TeleShake 95 platform mixer). The cassette was then washed three times in PBST-P using a 96-well microtiter plate washer (BioTek Instruments, Inc., Winooski, VT). Peptide-bound serum antibodies were detected using either 4.0 nM goat anti-human IgG (H+L) conjugated to AlexaFluor 555 (Invitrogen–Thermo Fisher Scientific, Inc., Carlsbad, CA) or 4.0 nM goat anti-human IgM (H+L) (Novus Biologicals, Centennial, CO), conjugated to DyLight 550 in secondary incubation buffer (0.5% casein in PBST-P) for 1 h with mixing at 37°C. After the final incubation, slides were washed three times with PBST-P followed by distilled water to remove residual salts. Slides were then sprayed with isopropanol and dried by centrifugation.

### Peptide microarray data extraction

Dried slides were imaged using an ImageXpress imaging system to detect fluorescently labeled secondary antibodies. The imager used an LED light engine (SemRock) centered at 532-nm wavelength to excite fluorophore-conjugated secondary Ab. Mapix (version 7.2.1; Innopsys, Carbonne, France) was used to place a grid alignment file over the obtained images and extract the median foreground pixel intensities using the central 60% of each feature.

### Data quality checks

Images were inspected to identify arrays with artifacts and image anomalies. The samples associated with such arrays were re-assayed on arrays from the same production batch as the original assay.

### Modeling of peptide binding using machine learning

Predictive models were built using machine learning methods based on feed-forward, backpropagating fully connected neural networks, similar to those described previously (36, 42). The peptide sequence was one-hot encoded by transforming each peptide into a vector of length 190. The vector length was derived from a maximum peptide length of 10 residue positions with 19 possible amino acids for each position. Feed-forward neural networks were built individually for each donor using R (version 4.2.2, R Foundation for Statistical Computing, Vienna, Austria) as the programming language and utilizing TensorFlow (version 2.11.0) and Keras (version 2.11.1) as the interface packages. The NN models were constructed using three hidden layers with 100 nodes each with a 10% dropout and no layer bias. Rectified linear unit (RelU) activation was used for each layer. Each NN model was trained 10 times using a random 90:10 split of the dataset each time. The data points were weighted by the frequency of peptides appearing in an intensity interval. To this end, the entire intensity range was subdivided into 100 equal bins, and the number of peptides falling into each bin was calculated. The weight for each peptide was computed using the following formula:


(1) $$w_{i}=\frac{1}{\sqrt{n_{i}}}$$

where *w_i_* is the weight of the *i*th peptide and *n_i_* is the number of peptides in the bin that the *i*th peptide falls into.

The accuracy of the model to predict Ab binding to the array was evaluated by predicting the binding to the held-out 10% of the data and reported as Pearson’s correlation between the measured and predicted binding intensities. Binding to *B. burgdorferi* epitopes was accomplished by applying the NN models to the *B. burgdorferi* B31 reference proteome (UniProt Accession # UP000001807) that had been represented as 10-mers with a sliding window of one amino acid offsets.

### Classification

Random forest decision trees with XGBoost were used to train classifiers for distinguishing patients from the different cohorts used. Each classifier model was trained 10 times on randomly selected 90% of the patients from the corresponding cohorts, and its performance was assessed on the remaining 10% of patients. All training steps and mean receiver operating characteristic curve calculations were performed in R.

### Outlier sum statistics

The outlier sum statistics was implemented following the method published by Tibshirani et al. (45). The predicted binding intensity values were first z-score normalized using the following:


(2) $$I_{i,j}^{z-norm}=\frac{I_{i,j}-mean(I_{i})}{SD\left(I_{i}\right)}$$

where *I_i_*,*_j_* is the predicted binding intensity value of the *j*th tile in the *i*th sample, and mean and SD are the mean and standard deviation values of *I_i_*, respectively. In this way, binding intensity values were all normalized to their corresponding mean and standard deviation values. Next, the Z-scores of the binding intensity values of the LD+ and LD− samples (“cases”) were calculated using the means and SD values of the LAD samples (“controls”):


(3) $$I_{i,j}^{diff}=\frac{I_{i,j}^{z-norm}-mean\left(I_{j\;\left(c\right)}^{z-norm}\right)}{SD\left(I_{j\;\left(c\right)}^{z-norm}\right)}$$

where *I_j_*_(_ *_c_*_)_ denotes the predicted binding intensities of the *j*th tile in the control samples. Afterward, a sliding window smoothing with a window size of 5 was applied to the data. Next, for each protein from the *B. burgdorferi* proteome, the tile with the maximum $I_{i,j}^{diff}$ value for each patient in the case cohort was determined. As a result, each protein is represented as its maximum binding value normalized against the control samples as a reference. These maximum binding values were then used to compute the outlier sum (OS) statistics for each protein:


(4) $$OS_{p}=\sum_{i=1}^{N}\begin{array}{c} I_{i}^{p},\;I_{i}^{p}>q_{0.75}\left(I_{\;}^{p}\right)+IQR\left(I_{\;}^{p}\right)\\ \;0,\;otherwise \end{array}$$

where *N* is the number of samples in the case cohort, $I_{i}^{p}$ represents the maximum binding value for protein *p* in sample *i*, $I_{\;}^{p}$ is the maximum binding value of protein *p* of the samples in the case cohort, and *q*_0.75_ and IQR are the third quartile and interquartile range of $I_{i}^{p}$ , respectively. The p-values for each $OS_{p}$ were calculated using the t-test, and a null distribution was obtained by randomizing the cohort assignments of the samples 1,000 times. The false discovery rate was calculated using the Benjamini–Hochberg adjustment for multiple comparisons.

### Bead-based assays and data analysis

The functionalization of Luminex MagPlex (Diasorin, Madison, WI) microspheres (beads) was performed by reacting the carboxylic residues of the microspheres and amine groups of proteins using 1-(3-(dimethylamino)propyl)-3-ethyl-carbodiimide hydrochloride (EDAC)/NHS chemistry. Briefly, 236 μL of each address of stock Luminex MagPlex microspheres (1.27 × 10^7^ beads/mL) was resuspended into 764 μL deionized water (DW; 18.2 MΩ·cm), followed by washing with 1 mL of DW. A total of 46 different regions of microspheres each representing a different spectral region for multiplex detection were used for the binding assays. The carboxylic residues of the microspheres were activated by incubating with 90 μL of 50 mg/mL of NHS (Sigma-Aldrich, St. Louis, MO) and 90 μL of 50 mg/mL of EDAC (Sigma-Aldrich) in 1 mL of 0.1 M sodium phosphate (Sigma-Aldrich) buffer for 20 min at room temperature (RT) under gentle rotation. For each bead region, the reaction using 106 microspheres and 5 μg of protein was carried out in 900 μL of 50 mM of MES (pH 5.0) buffer (Avocado Research Chemicals Ltd., Heysham, Lancashire, UK) for 2 h with gentle rotation at RT. After removing the supernatant, the functionalized microspheres were resuspended into 1 mL of PBS-TBN [PBS buffer (Life Technologies, Burlington, ON, Canada) with 0.02% Tween-20 (Sigma-Aldrich), 0.1% bovine serum albumin (BSA; Sigma-Aldrich), 0.02% sodium azide (Sigma-Aldrich), 150 mM sodium chloride (Life Technologies), and 50 mM sodium phosphate monobasic, pH 7.4]. The surface was then blocked with 1% BSA by rotating for 30 min at RT. The beads were washed three times with PBST. Next, all 46 regions of the functionalized microspheres were combined and mixed with 54 mL of PBST-BSA [PBS buffer with 0.1% Tween-20 (Sigma-Aldrich) and 1% BSA] buffer for further use. Microspheres functionalized with the VlsE protein served as positive control for the LD+ cohort, and “blank” beads that went through the same preparation steps, but were not functionalized with a protein, served as negative control. All washing and supernatant removal steps were performed using a MagJET separation rack (Thermo Fisher Scientific, Carlsbad, CA) to separate the microspheres from the solution.

For assays, 50 μL of functionalized microspheres at a concentration of 40 beads/μL (a total of 2,000 beads) was first dispensed into each well of 96-well a non-binding 96-well plate with a flat bottom (Corning, Corning, NY) using a Bravo automated liquid dispensing system (Agilent, Santa Clara, CA). This step was followed by incubation with 50 μL of serum sample (diluted at 1:500 in PBST) for 1 h at 37°C with shaking at 500 rpm. After washing three times with a magnetic microplate washer (Biotek 405 TS, Agilent), 100 μL of goat anti-human IgG secondary antibody (Jackson ImmunoResearch, West Grove, PA) diluted to 1:125 was added to each well and incubated for 30 min at RT with shaking at 500 rpm. After incubation, the beads were washed three times and resuspended in 100 μL PBST buffer. Binding signal intensities were then measured using a Luminex™ xMAP™ IntelliFlex (Diasorin) system.

The binding intensity values for each sample measured in the bead-based assays were normalized by computing the ratio between the intensity values and the intensity of a reference protein. The reference protein (O51141) was selected as a protein having the lowest CV when measured across all cohorts. The assumption was made that such a protein is least affected by the cohort-specific differences in antibody repertoires and thus can be used as a reference to compare binding intensities across patients.

## Data Availability

The original contributions presented in the study are publicly available. This data can be found here: https://figshare.com/articles/dataset/SI_table4_raw_array_data_csv/28299398.
